# Prioritizing Emerging Zoonoses in The Netherlands

**DOI:** 10.1371/journal.pone.0013965

**Published:** 2010-11-15

**Authors:** Arie H. Havelaar, Floor van Rosse, Catalin Bucura, Milou A. Toetenel, Juanita A. Haagsma, Dorota Kurowicka, J. (Hans) A. P. Heesterbeek, Niko Speybroeck, Merel F. M. Langelaar, Johanna W. B. van der Giessen, Roger M. Cooke, Marieta A. H. Braks

**Affiliations:** 1 National Institute for Public Health and the Environment, Bilthoven, The Netherlands; 2 Utrecht University, Utrecht, The Netherlands; 3 Delft University of Technology, Delft, The Netherlands; 4 Wageningen University and Research Centre, Wageningen, The Netherlands; 5 Erasmus Medical Centre, Rotterdam, The Netherlands; 6 Institute of Tropical Medicine, Antwerp, Belgium; 7 Institute of Health and Society, Université Catholique de Louvain, Brussels, Belgium; Columbia University, United States of America

## Abstract

**Background:**

To support the development of early warning and surveillance systems of emerging zoonoses, we present a general method to prioritize pathogens using a quantitative, stochastic multi-criteria model, parameterized for the Netherlands.

**Methodology/Principal Findings:**

A risk score was based on seven criteria, reflecting assessments of the epidemiology and impact of these pathogens on society. Criteria were weighed, based on the preferences of a panel of judges with a background in infectious disease control.

**Conclusions/Significance:**

Pathogens with the highest risk for the Netherlands included pathogens in the livestock reservoir with a high actual human disease burden (e.g. *Campylobacter* spp., *Toxoplasma gondii*, *Coxiella burnetii*) or a low current but higher historic burden (e.g. *Mycobacterium bovis*), rare zoonotic pathogens in domestic animals with severe disease manifestations in humans (e.g. BSE prion, *Capnocytophaga canimorsus*) as well as arthropod-borne and wildlife associated pathogens which may pose a severe risk in future (e.g. Japanese encephalitis virus and West-Nile virus). These agents are key targets for development of early warning and surveillance.

## Introduction

Human health is threatened by a wide variety of pathogens transmitted from animals to humans. In the Netherlands, a systematic approach for early warning and surveillance of emerging zoonoses and a blueprint for an efficient network of collaborators from the medical and veterinary professions to prevent and control emerging zoonoses are being developed by a consortium of national institutes for human and animal health (the EmZoo consortium). To support this task, a prioritized list of emerging zoonotic pathogens of relevance for the Netherlands was needed. The HAIRS Group in the UK [1] has developed qualitative decision trees to assess the zoonotic potential of emerging diseases [2] and to classify the risk to public health, based on probability and impact of infection [3].

Priority setting is a multi-dimensional problem, in which technical information is often intertwined with value judgments. Traditionally, a priority setting procedure entails asking a limited number of experts to reach consensus. An example of this approach in the domain of emerging zoonoses has been published in France [4]. This method is relatively straightforward, but not very transparent and the repeatability is low. Currently, semi-quantitative methods are frequently used in which criteria are divided into a limited number of classes (e.g. low, medium and high). Criteria may also be scored on arbitrary scales (e.g. 0, 1, …, 5), while scores for all criteria are aggregated to produce an overall score. An example of this approach was published in Belgium [5], and a similar approach was taken for animal diseases by McKenzie *et al.* [6] in New Zealand. Here, the transparency and the repeatability are improved, but the classes are chosen rather arbitrarily. Linear relations between the different classes of a criterion or between criteria are often assumed but are not supported by data. For the current project, the aim was to develop a quantitative method to rank emerging zoonoses using clearly interpretable criteria, expressed on natural numerical scales. Furthermore, weights were incorporated for these criteria, elicited by a systematic procedure from a panel of judges, independent from the authors or scientific experts in the project. The method was designed to simultaneously be the basis of a web-based knowledge management system.

The quantitative method is based on the well-established multi-criteria analysis (MCA) method. This method has been used in many decision making contexts including animal health [7]. MCA offer methods and techniques to structure complex decision-making. After completing the different phases, information can be introduced or modified without the necessity to completely redo the analyses. This is especially valuable in the priority setting of emerging zoonoses, where information changes constantly. In our approach to MCA, we combined objective information on the epidemiology and societal impact of zoonotic pathogens with subjective information on the relative weights of different criteria. The objective information was based on scientific evidence, while for the subjective information the values of individuals involved in the control of infectious diseases were sought.

## Methods

### Selection of pathogens

Zoonoses are defined as diseases that can be transmitted between vertebrate animals and man under natural conditions. An emerging zoonoses is a zoonosis that is newly recognized or newly evolved, or that has occurred previously but shows an increase in incidence or expansion in geographic, host, or vector range [8]. Of 1415 known species of human pathogens, there are 868 zoonotic pathogens [9], but only a limited number of them is considered relevant as emerging zoonoses for the Netherlands.

Information from recent published studies on emerging zoonoses in the Netherlands [10] and from other European countries [4], [11], [12], [13], [14], [15] was taken into account. Furthermore, relevant information was gathered from signals of emerging zoonoses from internet sources of public health and veterinary organizations including the WHO, OIE, HPA and CDC and ProMED-mail. In addition, expert members of the Emzoo consortium were invited to suggest additional pathogens. This process resulted in a long-list, including all pathogens (174) mentioned as emerging zoonoses in one of the sources mentioned above. Only pathogens with a proven zoonotic potential [2] were included in our final list. To condense the resulting long-list to a more manageable short-list, five additional decision rules were applied. A zoonotic pathogen was excluded from the list if:

- non-human primate species form its only known reservoir. These reservoir species are not likely to occur as free ranging species in Europe and the pathogens have little public health significance other than very specific occupational risks, e.g. Simian foamy virus;
- its specific only known reservoir species is absent in Europe, e.g. Sin nombre virus;
- its vector (in case of a vector-borne zoonotic pathogen) family (not vector species) is absent in Europe, e.g. *Trypanosoma* spp.;
- the zoonotic aspects involved a single species jump, after which the pathogens further evolved and became effectively and essentially transmissible from human to human e.g. new influenza H1N1 or HIV.

This analysis finally resulted in a short-list of 86 emerging zoonotic pathogens of relevance for the Netherlands (see database in Annex S2), which are evaluated by the risk-ranking method.

### Listing and structuring of criteria

**Figure 1:**
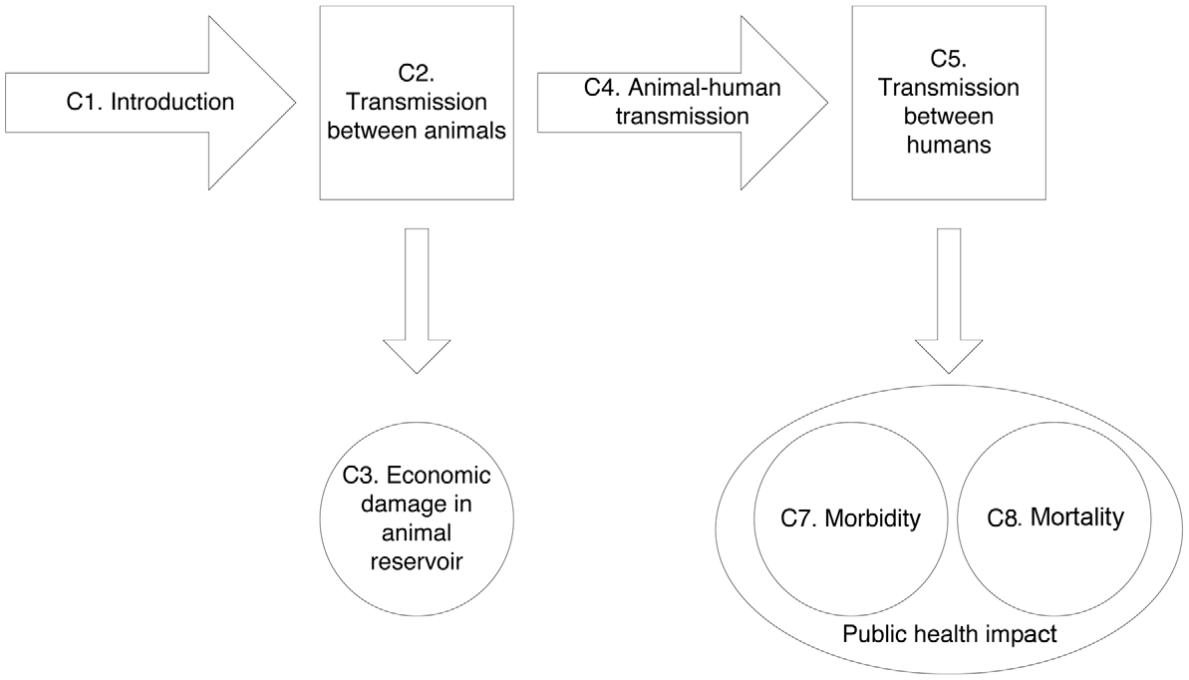
Flow chart of the pathway from introduction of a zoonotic pathogen to public health impact, represented by 7 criteria (C1–C7) from which the risk to public health of emerging zoonoses was derived.

We quantified the risk to public health of emerging zoonoses by applying seven criteria that covered the complete pathway from introduction to societal impact (Figure 1). All criteria were scored on a natural scale, and were divided into 4-5 levels; often covering several orders of magnitude in terms of effects (see Table 1 and Annex S1). For subsequent analysis, each class was represented by a point estimate, representing a central value in the range.

**Table 1:** Quantifying criteria to assess risk of emerging pathogens.

Criterion	Description	Unit	Levels	Value (*x*)	Scaled value (*x′*)*	Transformed value (*X*)*
C1	Probability of introduction into the Netherlands	% / year	<11–910–99100	0.5550100	0.0050.050.51	0.0000.4350.8691.000
C2	Transmission in animal reservoirs	Prevalence per 100,000 animals	<11–100100–1,0001,000–10,000>10,000	0505005,00050,000	0.00000010.000050.00050.0050.1	0.0000.3860.5280.6710.857
C3	Economic damage in animal reservoirs	Million euro per year	<11–1010–100>100	0.5550500	0.00050.0050.050.5	0.0000.3030.6060.909
C4	Animal-human transmission	Prevalence per 100,000 humans	1–100100–1,0001,000–10,000>10,000	505005,00050,000	0.000050.00050.0050.1	0.0000.2330.4650.767
C5	Transmission between humans	Prevalence per 100,000 humans	<11–100100–1,0001,000–10,000>10,000	0505005,00050,000	0.00000010.000050.00050.0050.1	0.0000.3860.5280.6710.857
C6	Morbidity (disability weight)	None	<0.030.03–0.10.1–0.3>0.3	0.020.060.20.6	0.020.060.20.6	0.0000.2810.5890.869
C7	Mortality (case-fatality ratio)	%	00–0.10.1–11–1010–100	00.050.5550	0.00000010.00050.0050.050.5	0.0000.5280.6710.8140.957

*Point estimates *x* were first scaled (*x′*) between 0 (best possible option) and 1 (worst possible option). C1, C6 and C7 are naturally bounded between 0 and 1; for C2, C4 and C5 a worst possible option of the prevalence of 100,000 per 100,000 was used. For C3, a worst possible option of 1,000 M€ was used. Best possible options of 0 were replaced by 0.0000001. Subsequently, transformed scores were calculated as *X* = 1−log(*x′*)/log(*x′*_ref_), where *x′*_ref_ is the scaled score for the best possible option.

### Evaluating pathogens on the selected criteria

Where possible, levels were assigned to pathogens based on published literature. Values were to reflect the current situation in the Netherlands, given the existing level of prevention and health care including vaccination and infrastructure (water supply, sewerage, food safety controls) *et cetera*. We, therefore, mainly used data from industrialized countries. For many pathogens currently available data were insufficient, and in those cases we tried to evaluate criteria using simple decision rules. In the absence of both sufficient data and decision rules, expert opinion was employed and related uncertainty was expressed by assigning a pathogen to more than one level. All assignments were made from the societal perspective, i.e. the impact on all affected parties and sectors of economy was considered.

### Determining the weight of each criterion

Weights were based on panel sessions with different groups of participants, representing different professional groups involved in infectious disease control:

- Risk managers from the Dutch Ministries of Agriculture and Public Health (n = 7);
- Infectious disease specialists from medical microbiological laboratories and from regional public health services (n = 11)
- Students in the medical and veterinary faculties of Utrecht University (n = 11).

Each panel session started with an explanation of the objectives and approaches of the project. Panel members were invited to comment on the approach and ask questions about any aspect. Discussion was specifically stimulated on the criteria and their scores, as ranking these was the core task of the panel members.

**Figure 2:**
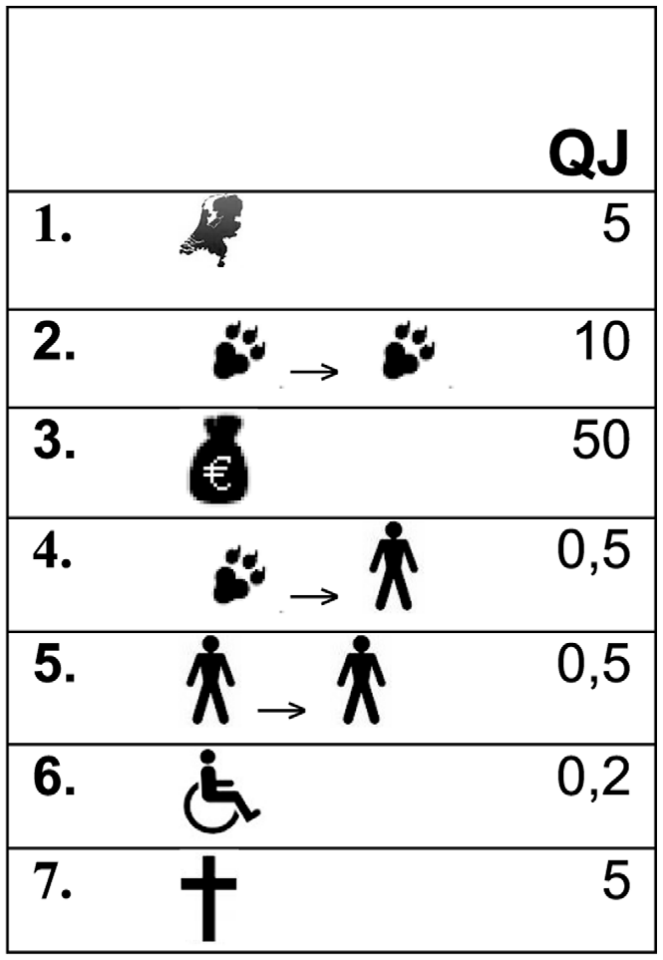
Example of card of a randomly generated scenario (QJ) used in the panel session to determine the relative weights of criteria. The numbers 1–7 represent the criteria C1–C7 (for details see Table 1).

For the ranking exercise, five groups of seven scenarios were generated. Each scenario (designated by a two letter code, e.g. QJ) represented a hypothetical zoonotic agent, by randomly choosing a level for each criterion, subject to certain constraints: scenarios were chosen as not to ‘majorize’ each other (i.e. no scenario should have a higher risk level on all criteria than any other in the same set), and implausible scenarios (i.e. with low animal prevalence yet very high costs) were omitted. Each scenario was presented to the panel members on a small card (Figure 2). Panel members were asked to place the scenario that they considered to represent the lowest risk to the left of their table and the highest risk scenario to the right. They were then asked to arrange the remaining five scenarios in between these two extremes, in order of increasing risk. To alleviate potential effects of training and fatigue, the five groups of seven scenarios (denoted by G1, …., G5) were offered to one half of the panel members in the order G1, G3, G5, G4, G2 and to the other half in the order G3, G2, G4, G1, G5. Data were entered in a Microsoft Excel spreadsheet independently by two analysts, and any discordance was resolved by referring to the original data sheets.

Panel rankings were checked for consistency in two ways. Firstly, scenario group G2 included two scenarios that also occurred in G1, G3 contained two scenarios from G2 and so on. Consistency was evaluated by calculating the number of pairs that were ranked differently (with a maximum of 4). Secondly, all panel members received G2 again by (e-)mail two weeks after the session and were asked to re-rank the scenarios. Results were considered inconsistent if the rank of a scenario shifted two or more positions, and the number of inconsistencies (with a maximum of 30) were counted.

Data-analysis was carried out by probabilistic inversion, as described by Kurowicka *et al.* [16]. Further technical details on probabilistic analysis as a method to model stakeholder preferences can be found in Nesloo and Cooke [18]. Detailed results and software code used for this particular project can be obtained from one of the authors (d.kurowicka@ewi.tudelft.nl). Probabilistic inversion consisted of the following steps:

- Evaluation of randomness.
- Transformation of values (Table 1).
- Optimization of constraints.
- Main analysis (probabilistic inversion)

A simpler method to prioritize infectious diseases for surveillance was proposed by Krause *et al.* [17]. To compare with our approach to elicit preference-based weights, panel members were also asked to directly assign a rank order to the seven criteria and mean ranks were calculated.

### Aggregation of data

A linear model was applied, which combined the mean weights from the panel session with transformed values for all 86 zoonotic agents. The model calculates the score *S_i_* of a pathogen as:
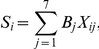
where *X_ij_* is the (transformed) value assigned to pathogen *i* on criterion *j* and *B_j_* is the weight of criterion *j*.

These results were then normalized to a value between 0 and 1 by calculating the scores for the pathogen with the highest and lowest theoretical risk (i.e. for which the values on all criteria were at the highest or the lowest level).

Uncertainty in the transformed scores was included as discrete distributions with equal weights, and quantified by Monte Carlo simulation in @RISK Professional Version 5.0 (Palisade Corporation, Ithaca, NY USA), an add-in to Microsoft Excel.

### Sensitivity analysis

To assess the impact of different model assumptions on the outcomes, several alternative scenarios were evaluated. These included:

- Equal weights. Instead of using the preference-based weights from the panel sessions, each criterion was assigned an equal weight.
- Semi-quantitative method. Instead of assigning a transformed value to each level as shown in Table 1, values of 1 … 5 were assigned to all criteria. Scores were calculated using equal weights.
- Deterministic model. An interactive website (Emerging Zoonoses Information and Priority system (EZIPs; http://ezips.rivm.nl) was developed that allows the user to change scores for any pathogen on each criterion to evaluate the possible impact of uncertain or modified information. It is also possible to exclude one or more criteria from the ranking, to compute scores with equal weights or to introduce a new pathogen and compare it with pathogens already in the database. For technical reasons, a stochastic model could not be implemented in the website and, therefore, uncertain values were replaced by single estimates. Single estimates were chosen so that the score was as close to the mean score from the stochastic model as possible. However, as there are only few levels per criterion, deviations could not be avoided. In addition to the results of the MCA, the website also contains descriptive information on all pathogens in 5 categories: Taxonomy, Human and Animal Disease, Reservoirs, Transmission, and Geographical distribution.

### Cluster analysis

Policy makers may to better grasp a categorization of diseases when expressed in qualitative terms (low, middle and high importance), than when expressed as a continuous number. We therefore implemented a cluster analysis. Based on an adapted version of the methodology used in Cardoen et al. [5], groups of different importance were identified by Classification and Regression Tree analysis (CART Version 6.0, Salford Systems, San Diego, California, USA [19]). As the normalized score is a continuous variable, we aim to obtain subgroups with minimal within group variance (grouping zoonoses with similar importance). Starting with all the pathogens the method will in first instance obtain a binary split into two groups (nodes) that are most homogeneous with respect to the normalized score. The two subgroups will then be further split so that the “purest” subgroups are obtained. The process is then continued until the nodes can not be further “purified” using a technique called cross-validation [20]. In contrast with [5], we did not use the mean total scores per disease (i.e. one value per disease) as input, but the output of the Monte Carlo simulations. This accounts for the existing uncertainty in the normalized scores. The categorical variable comprising the names of the pathogens was used as a discrimination variable. In this way, Monte Carlo samples of the same pathogen were kept together in the different clusters of pathogens.

## Results

### Listing and structuring of criteria

Details of criteria are given in Table 1, a full description can be found in Annex S1, including decision rules for assigning levels in absence of data.

### Evaluating pathogens on the selected criteria

A full table of scores of criteria of each of the 86 pathogens is presented in Annex S2.

### Determining the weight of each criterion

An example of a group of randomly generated scenarios that were ranked in panel sessions is presented in Table 2. The consistency between ranking in the panel session and the repetition after two weeks was good: 11 panel members did rank the scenarios in the same order in both sessions, and 10 provided only one answer that was not consistent with the previous ranking. 6% of scores resulting from ranking the same group after two weeks were considered inconsistent, and no panel member scored more than 20% inconsistencies. It was concluded that scores were sufficiently consistent to warrant further analysis. The results for group 1 (G1) are given in Table 3 as an example. Scenarios GF and WL represent the highest risk by the panel's opinion, while NW and QJ are considered to represent the lowest risk. Scenario VG is ranked as of medium risk, and there is considerable disagreement between the panel members on the risk of scenarios JR and ZC.

**Table 2:** Example of randomly generated scenarios (Group 1).

Code	QJ	VG	GF	JR	ZC	WL	NW
C1	5	50	50	0.5	50	50	50
C2	10	0.5	10	0.05	0.5	0.5	0.5
C3	50	50	5	50	50	50	50
C4	0.5	0.05	0.5	0.5	0.05	10	0.05
C5	0.5	10	0.5	10	0.05	0	0.05
C6	0.2	0.6	0.02	0.2	0.6	0.06	0.2
C7	5	0.5	50	50	5	50	0.5

The Table shows the code names of the seven randomly generated scenarios (QJ, VG, …) and the values assigned to each of the seven criteria (C1–C7, for details see Table 1).

**Table 3:** Example of results of ranking random scenarios within Group 1.

Rank	1st	2nd	3rd	4th	5th	6th	7th
QJ	2	**9**	**11**	4	2	0	1
VG	0	0	**5**	**7**	**11**	3	3
GF	0	0	0	**6**	**5**	**9**	**9**
JR	**7**	1	1	4	4	**7**	**5**
ZC	1	10	**8**	**6**	3	1	0
WL	2	1	1	1	4	**9**	**11**
NW	**17**	**8**	3	1	0	0	0

QJ-NW represent scenarios in Group 1 (see Table 2). 1^st^ rank represents the scenarios with the lowest risk while 7^th^ rank represents the scenarios with the highest risk. For example, scenario QJ was ranked as the lowest risk by 2 panel members. All rows and columns add up to 29, the total number of participants.

Results in bold (greater than 4) remain after elimination of weak signals to reduce the number of constraints for probabilistic inversion; hence the number of constraints is reduced from 49 to 16.

Including all signals in the model in which four or more panel members ranked the scenario at a particular position in the analysis (as indicated in Table 3 for G1) resulted in 51 constraints to be taken into account from the combined dataset of G1, G2 and G5. The scores of two out of five groups were not significantly different from random ordering and these groups were excluded from further analysis. The linear model was sufficient to reproduce the panel members' preferences.

Table 4 shows, for each criterion, the weights obtained and their standard deviation. Based on rankings by panel members, probabilistic inversion identified the human case-fatality ratio and animal-human transmission the most important criteria, whereas they considered transmission between animals, human morbidity and economic damage in animals least important. The coefficient of variation (standard deviation / mean) varied between 14 and 28%, reflecting deviating opinions between panel members about the relative importance of criteria.

**Table 4:** Comparison between preference-based weights (this paper) and direct ranking [17].

	Preference-based weights		Direct ranking
	Mean weight	SD	Mean rank
C1	0.418	0.100	4.14
C2	0.292	0.040	2.41
C3	0.337	0.069	1.41
C4	0.626	0.103	5.22
C5	0.339	0.096	5.29
C6	0.181	0.028	4.45
C7	0.643	0.113	5.24

Table 4 compares the weights derived by probabilistic inversion with the simple ranking method as proposed by Krause *et al.* The participants consider C5, C7 and C4 as the more important criteria when they rank them directly but the probabilistic inversion excludes C5 as important criterion. There is no significant correlation between both methods (p = 0.29, linear regression).

### Aggregation of data

**Figure 3:**
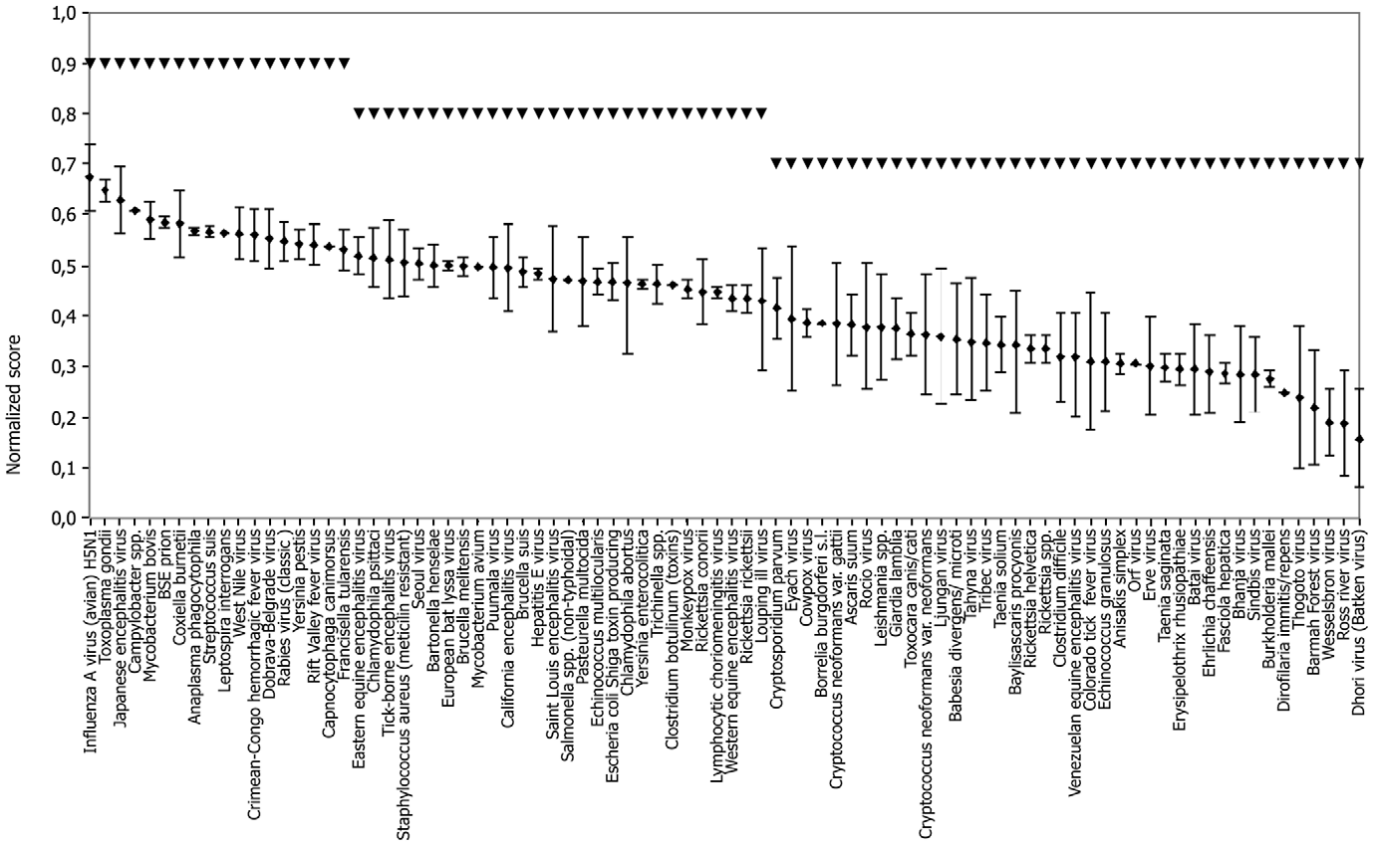
Emerging zoonotic pathogens relevant for the Netherlands (x-axis), prioritized according normalized scores (y-axis, means and 90% confidence intervals based on Monte Carlo simulation). Three groups of statistically different importance were identified by Classification and Regression Tree analysis and are represented by dashed lines. Mean (standard deviation) of the full dataset: 0.423 (0.124). Mean (standard deviation) of the three clusters: 0.577 (0.047); 0.476 (0.044); 0.317 (0.083).

Figure 3 shows the results of combining in the linear model the levels per pathogen with the mean weights as described above. The confidence intervals reflect the valuations of a random stakeholder, given uncertainty on criteria levels of the zoonoses. The model appears to have good discriminative power. Within the possible range for normalized scores of 1 to 0, there is a rather continuous decrease in normalized scores from 0.68 for the pathogen with the highest risk (Influenza A virus (avian) H5N1) to 0.15 for the pathogens with the lowest risk (Dhori virus). The error bars around the normalized scores reflect uncertainty about the epidemiological characteristics of the pathogens, which is particularly large for many exotic viruses. Note however that the uncertainty tends to be greater for pathogens with lower normalized scores. Inspection of Annex S2 shows that the greatest uncertainty was associated with criteria relating to transmission in the animal reservoir (C2) and from animals to humans (C4). There was little uncertainty in the transmission between humans (C5).

### Sensitivity analysis

**Figure 4:**
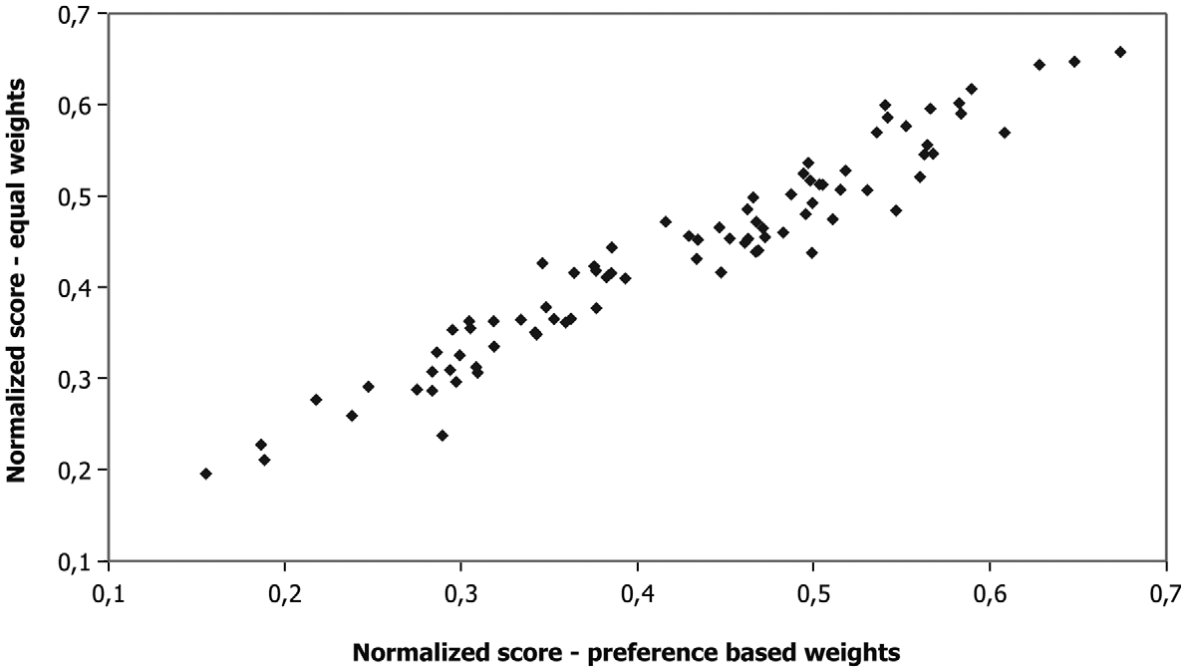
Comparison of normalized scores using preference-based weights and equal weights.

Figure 4 shows relatively good correlation between scores obtained with the baseline model using preference-based weights and an alternative model in which each criterion is given equal weight. Yet, even relatively small differences in scores may significantly affect the ranking of pathogens.

**Figure 5:**
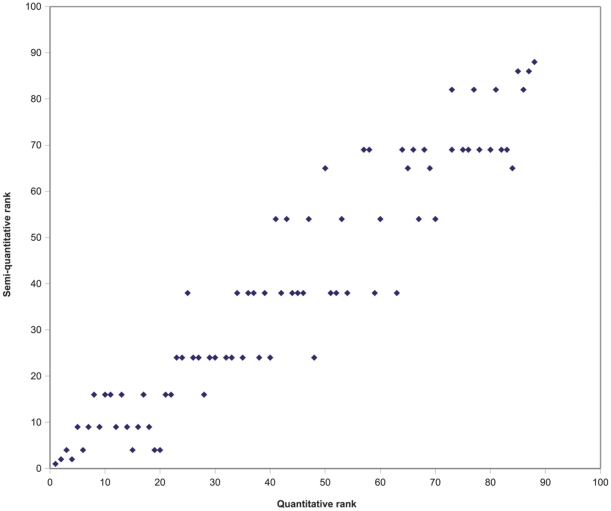
Comparison of ranking using quantitative and semi-quantitative model.

A comparison between the quantitative method proposed in this paper and the semi-quantitative method currently used by many authors (both models with equal weights) showed that despite a general tendency for ranks to increase in parallel, the discriminative power of the quantitative method was much larger. The semi-quantitative method can only assign a discrete number of scores, whereas the quantitative method uses the full scale in a continuous manner. Rankings according to both methods may also be quite different (Figure 5). Most pathogens were ranked from five places lower to 15 places higher, but extremes from 16 places lower to 25 places higher did occur.

### Cluster analysis

Three statistically different groups of importance were identified by CART and are indicated by (dashed) lines in Figure 3. The optimal number of subgroups was 29, but for the sake of practical use of the results, we report the three main clusters only. The clusters comprise 18, 28 and 40 pathogens, respectively. Splitting the tree further in e.g. five clusters subdivided the cluster with the lowest normalized scores and hence is not very informative for risk management purposes.

Among the first cluster including 18 pathogens with the highest normalized scores, there are one prion, 7 viruses, 9 bacteria and one protozoan parasite. 8 are already present in the Netherlands while 10 are not. Helminths are not represented in this group. The obtained grouping is not very sensitive to the number of Monte Carlo simulations. Indeed, the grouping obtained with 400 and 200 Monte Carlo simulations only differed by one pathogen shifting from one group to another. No difference was noted between 400 and 600 Monte Carlo simulations, indicating that 600 simulations were more than sufficient for a robust grouping.

## Discussion

We describe a quantitative, stochastic method to rank the risk of emerging zoonotic pathogens for the Netherlands. The approach differs from several previously published methods. We decided to restrict the number of criteria. With higher numbers, it becomes increasingly complex to develop validated databases in which pathogens are assigned to multiple possible values. Furthermore, choosing between different scenarios as in our panel studies becomes less meaningful as respondents will only use a limited number of criteria to base their judgment on. By choosing criteria at a high level of integration, we do, however account for many criteria that are used in similar exercises, either explicitly by incorporating them in decision rules or implicitly in the transmission criteria.

In contrast to most current approaches, we scored our criteria using associated numerical scales, rather than non-informative *ad-hoc* scales. This forces explicit consideration of the available scientific evidence and we suggest that our quantitative approach is less arbitrary in assigning values to possible levels that a criterion can take, and is therefore more realistic than a semi-quantitative approach. Our comparison with currently used semi-quantitative methods (Figure 5) shows that there are considerable differences between the quantitative and semi-quantitative approach. We also introduce preference-based weights in the calculation of the pathogen scores. The weights are reflecting the preferences of a panel of decision makers, in our case professionals involved in infectious disease control. Our comparative analysis shows that using weights does affect ranking, but to a lesser extent than introducing numerical scales. We also found that our elaborate method of establishing weights through choice experiments provided weights that were very different from those obtained with a simple ranking exercise.

Assigning levels to the 86 pathogens on the short-list was found to be a difficult process that required several iterations involving literature studies and evaluation by pathogen-specific experts. Nevertheless, considerable uncertainty remains, part of which was expressed in uncertainty ranges around the normalized scores. By identifying the factors that contribute most to the uncertainty in quantified risk for pathogens with high normalized scores, these results can be used to prioritize additional data collection and analysis. The current method can easily be updated to incorporate new data in a transparent way. Furthermore, the web tool allows all users of the system to explore the impact of different value assignments in an interactive mode.

The pathogens with the highest score according to the baseline model would be proposed as priorities for risk management activities. Subdivision into smaller groups with different implications for risk management is suggested. This is illustrated by considering the 18 pathogens in the cluster with the highest normalized scores. A major subdivision is between pathogens already established in the Netherlands and pathogens that are not. Surveillance and risk management strategies are likely to be different for these categories. As a next step in the EmZoo project, all pathogens were evaluated for the availability of hum and and veterinary diagnostic methods, and surveillance systems. Results showed that many gaps in diagnostics and surveillance exist, also for the zoonoses in the first cluster. It was suggested that many of these gaps can be complemented by developing generic surveillance systems, which, in an efficient way, monitor for more than one pathogen at a time. Thus, the development of mosquito monitoring, tick monitoring, rodent monitoring, and syndromic surveillance in humans and horses was recommended.

Instead of using expert panels, the same method could also be used to identify issues that are important for the general public (citizens) as their weighing of criteria could be different. These results might offer opportunities to improve risk communication to the general public. Moreover, the method could also be used in another context (e.g. in developing countries) in order to prioritize pathogens that should be addressed in developmental aid programmes.

The model for priority setting presented here is based on criteria reflecting the epidemiology and societal impact of zoonotic diseases. Risk perception by the general public is not included in this model, but may pose additional challenges to policy makers. Further work to include risk perception as a second dimension in the priority model is recommended.

In summary, the EmZoo project has resulted in:

- the development of a cross-disciplinary network to deal with zoonoses threats;
- the development of systematic, explicit and quantitative estimates of risk; and
- a web-based knowledge management system.

## Supporting Information

Annex S1 Criteria: definitions, ranges, point estimates, and decision rules.
(0.07 MB DOC)

Annex S2 Database.
(0.06 MB XLS)
