# Supplementary material for: Prioritizing Emerging Zoonoses in The Netherlands
Source: PLoS One. 2010 Nov 15;5(11):e13965. doi: 10.1371/journal.pone.0013965 (PMC2981521; doi:10.1371/journal.pone.0013965)
Supplement: Annex S1 — Criteria: definitions, ranges, point estimates, and decision rules. (0.07 MB DOC) [file pone.0013965.s001.doc]

#### 1: Probability of introduction of the pathogen in the Netherlands

*Criterion*: Probability of introduction of pathogen.

*Definition:* This criterion describes the probability that a zoonotic pathogen will be introduced in the Netherlands in the following year. This probability depends on the introduction of an infected entity (infected reservoirs, vectors, human cases or food). Moreover, it depends on the prevalence of an infection in such an entity and the intensity in which those entities enter the Netherlands. The result depends on the type of entity in question.

*Ranges and point estimates:* The probability of introduction of a pathogen will be estimated using the decision rules described below appointing it to one of four probability intervals:

- < 1%, point estimate 0.5%
- 1-9%, point estimate 5%
- 10-99%, point estimate 50%
- 100%.

*Decision rules:*

Import of animals (farm animals, pets and exotics) and food

**< 1%:** The infection exists in the countries that export to the Netherlands, but these countries have an effective control program and/or the Dutch import control is effective;

**1-9%**: The infection does exist in the countries that export to the Netherlands but these countries do not have an effective control program, and the Netherlands do not have an effective program either or import only limited number of animals [< 1000/year] or food [< 1000 ton/year];

**10-99%**:The infection exists in the countries that export to the Netherlands, and these countries do not have an effective control program neither does the Dutch have an effective import control and many live animals [> 1000/year] or food [> 1000 ton/year] are imported;

**100%:** The infection exists among animals living in the Netherlands.

Wildlife species (including reservoirs and vectors):

**<1% :** Wildlife species do inhabit countries surrounding the Netherlands, but no infection has been found while it has been investigated;

**1-9%**: Wildlife species inhabit countries surrounding the Netherlands, but possible infections have not been investigated

**10-99%**:Wildlife species inhabit countries surrounding the Netherlands and an infection has been detected

**100%:** Infected wildlife species have been found in the Netherlands.

Humans:

**<1% :** Humans from endemic areas stay in the Netherlands less than one day (transit) or Dutch people travel to endemic areas;

**1-9%** : Humans from endemic areas stay in the Netherlands longer than one day ;

**10-99%**:Infection exists among humans inhabiting the Schengen countries;

**100%:** The disease is indigenous to the Netherlands. The presence of the pathogen is documented.

**2:. Transmission between animals**

*Criterion:* Fraction of animal reservoir infected.

*Definition:* This criterion describes the prevalence of infections in animal reservoirs.

*Ranges and point estimates:*

- <1 infections per 100,000 animals per year, point estimate 0%
- 1-100 infections per 100,000 animals per year, point estimate 0.05%
- 100-1,000 infections per 100,000 animals per year, point estimate 0.5%
- 1,000-10,000 infections per 100,000 animals per year, point estimate 5%
- >10,000 infections per 100,000 animals per year, point estimate 50%

*Decision rules:* None

**3: Economic damage in animal reservoir**

*Criterion*: Economic costs

*Definition:* This criterion describes the costs for the Dutch society given the discovery of an infection in the Dutch animal reservoir, and transmission between animals has occurred. The costs relate to the agricultural sector (production animal farms, suppliers, slaughter houses, and food industry) and the government. The costs include costs associated with control of the disease (culling, vaccination, compensation etc) and the costs of lack of occupancy of stables, loss of breeding animals, lost returns and the damage to the market through the loss of a share in the market for a long period of time and loss in the tourist industry. These costs depend on preceding criteria, because a zoonotic agent that also causes animal diseases and spreads quickly will demand more intense and expensive control measures.

*Ranges and point estimates:* The costs of the emerging pathogen will be estimated using the decision rules described below appointing it to one of four intervals:

- <1 M Euro per year, point estimate 0.5 M Euro per year
- 1-10 M Euro per year, point estimate 5 M Euro per year
- 10 – 100 M Euro per year, point estimate 50 M Euro per year
- >100 M Euro per year, point estimate 500 M Euro per year

*Decision rules:*

**<1 M Euro per year:** In the Netherlands, farm animals do not get ill or only a few animals get ill and control is done at the level of the animal itself.

**1- 10 M Euro per year**: In the Netherlands, farm animals can get ill and control is done at the level of the farm itself.

**10 – 100 M Euro per year**: In the Netherlands, farm animals can get ill and control is done at the level of the section or region.

**>100 M Euro per year**: In the Netherlands, farm animals can get ill and control is done at national or international level.

**4. Transmission from animals to humans**

*Criterion:* Fraction of humans infected by animal-human transmission.

*Definition:* This criterion describes the prevalence of infections in humans caused by infected.

*Ranges and point estimates:*

- 1-100 infections per 100,000 humans per year, point estimate 0.05%
- 100-1,000 infections per 100,000 humans per year, point estimate 0.5%
- 1,000-10,000 infections per 100,000 humans per year, point estimate 5%
- >10,000 infections per 100,000 humans per year, point estimate 50%

*Decision rules:* None

**5. Transmission between humans**

*Criterion:* Fraction of humans infected by human-human transmission.

*Definition:* This criterion describes the prevalence of infections in humans caused by human to human transmission.

*Point estimates:*

- <1 infections per 100,000 humans per year, point estimate 0%
- 1-100 infections per 100,000 humans per year, point estimate 0.05%
- 100-1000 infections per 100,000 humans per year, point estimate 0.5%
- 1,000-10,000 infections per 100,000 humans per year, point estimate 5%
- >10,000 infections per 100,000 humans per year, point estimate 50%

*Decision rules:* None

**6. Morbidity**

*Criterion*: Loss of health related quality of life

*Definition*: This criterion reflects the effect of the disease on the health related quality of life. The value of the criterion is anchored between 0 (full health) en 1 (worst possible health state) and depends on both the severity and the duration of the disease. For a large number of diseases such disability weights have already been published.

*Point estimates:* Four intervals for the morbidity are used

- disability weight < 0.03; point estimate 0.02
- 0.03 < disability weight < 0.1; point estimate 0.06
- 0.1 < disability weight < 0.3; point estimate 0.2
- disability weight > 0.3; point estimate 0.6

*Decision rules*: The scores are obtained by analogy of illnesses already in the list below.

| **Disease label** | **Duration (in days)** |
| --- | --- |
| ***Very mild (disability weight < 0.03)*** |  |
| Otitis media | 14 |
| Hepatitis | 30 |
| Folliculitis | 7 |
| Cystitis | 14 |
| Gastroenteritis, severe | 10-15 |
| Conjunctivitis | 7 |
| Tonsillitis | 7 |
| Bronchitis | 14 |
| ***Mild (0.03<disability weight < 0.1)*** |  |
| Allergic rhinitis | 119 |
| Reactive arthritis | 42 |
| Tinea pedis | 183 |
| Eczema | 35 |
| Otitis externa | 35 |
| Gastroenteritis, hospitalized | 7-14 |
| Laryngitis | 7 |
| Sinusitis | 183 |
| Irritable bowel syndrome | 183 |
| Haemolytic uremic syndrome | 30 |
| Visual disorder, mild | 365 |
| Hepatitis | 92 |
| Gastroenteritis, chronic | 183 |
| Influenza | 14 |
| ***Moderate (0.1<disability weight < 0.3)*** |  |
| Inflammatory bowel disorder | 183 |
| Reactive arthritis | 183 |
| Tuberculosis | 365 |
| Chronic pulmonary disease (bronchitis, asthma, emphysema) | 365 |
| Diabetes mellitus | 365 |
| ***High (disability weight > 0.3)*** |  |
| Renal failure | 365 |
| Guillain-Barré syndrome | 365 |
| Visual disorder, severe | 365 |
| Paraplegia | 365 |
| AIDS | 365 |
| Meningitis |  |
| Dementia | 365 |

In case a pathogen can cause more than one disease, or if there are vulnerable groups, a population weighted average is applied.

**7. Mortality**

*Criterion*: Case fatality ratio

*Definition:* This criterion describes the case-fatality ratio of the illness, which depends on the nature of the infection and the health status of the infected person.

*Point Estimates*: Five intervals are used:

- 0%
- 0-0.1%, point estimate 0.05%
- 0.1-1%, point estimate 0.5%
- 1-10%, point estimate 5%
- 10-100%, point estimate 50%

*Decision rules*: None.

In case a pathogen can cause more than one disease, or if there are vulnerable groups, a population weighted average is applied. This also implies that that if fatal cases only occur in vulnerable groups, the case-fatality ratio in that group should be multiplied by the relative size of the vulnerable group in the population.
